# Analysis of Inflammatory and Thyroid Hormone Levels Based on Hepatitis A and B Virus Immunity Status: Age and Sex Stratification

**DOI:** 10.3390/v16081329

**Published:** 2024-08-20

**Authors:** Hyeokjun Yun, Jae-Sik Jeon, Jae Kyung Kim

**Affiliations:** 1 Department of Medical Laser, Graduate School of Medicine, Dankook University, Cheonan-si 31116, Republic of Korea; 10621yhj@naver.com; 2 Department of Biomedical Laboratory Science, College of Health Sciences, Dankook University, Cheonan-si 31116, Republic of Korea; zenty87@naver.com

**Keywords:** C-reactive protein, free T4, hepatitis A antibodies, hepatitis B antibodies, thyroid-stimulating hormone

## Abstract

This study investigated the potential associations between hepatitis virus antibody status and thyroid and inflammatory function. The C-reactive protein (CRP), thyroid-stimulating hormone (TSH), and free thyroxine (FT4) levels were measured in individuals with and without antibodies to the hepatitis A virus (HAV) and hepatitis B virus (HBV). Participants were stratified by age, sex, and HAV/HBV antibody status. Participants with and without antibodies to HAV and HBV had normal CRP, TSH, and FT4 levels. However, notable discrepancies were observed in FT4 levels among participants with HAV antibodies and in CRP and FT4 levels among those with both HAV and HBV antibodies, suggesting potential associations between viral immunity and thyroid function, especially in younger participants. Significant variations in thyroid hormone levels were noted when the sample was stratified by sex and HAV and HBV antibody status, indicating that the association between antibody status and thyroid hormone levels varied by sex. This study underscores the need for further research on the effect of viral immunity on inflammatory parameters and thyroid hormone levels.

## 1. Introduction

Hepatitis A virus (HAV) and hepatitis B virus (HBV) infections pose significant health challenges worldwide. Both viruses can cause liver inflammation and affect various physiological functions [1,2,3,4]. HAV is a predominant factor involved in acute viral hepatitis worldwide and commonly spreads through the fecal–oral route or via contaminated food and water [2]. The presence of anti-HAV immunoglobulin G (IgG) antibodies indicates lifelong immunity to HAV. Since the early 1990s, highly efficient vaccines known for their swift seroconversion rates have been available, thus enabling effective prevention both before and after exposure. Immunity to HAV is typically recognized when IgG anti-HAV antibodies reach a titer exceeding 10–33 IU/L, depending on the vaccine manufacturer and immunoassays used, although the precise protective threshold against an HAV challenge has not been determined [2,5,6,7,8].

Antibodies against hepatitis B surface antigens (anti-HBs) play a vital role in conferring immunity against HBV and significantly influence the course of the infection. Anti-HBs serve as an indicator of recovery, with a positive response typically signaling effective infection control and improvement in the patient’s health [9]. Conversely, the absence of an anti-HB response over an extended period is an indicator of unfavorable disease outcomes. Hence, monitoring anti-HB levels is essential throughout the treatment and recovery phases to assess the patient’s progress [3,4,10].

Thyroid hormones, such as free thyroxine (FT4) and the thyroid-stimulating hormone (TSH), are essential for governing metabolism and sustaining general well-being [11,12]. Thyroid dysfunction is common in various chronic illnesses, including severe liver disease resulting from hepatitis. The evaluation of thyroid function depends primarily on measuring the levels of thyroid hormones (T3 and T4) and TSH, which triggers the TSH receptor [11]. Previous studies have shown a negative correlation between serum TSH levels and the severity of HAV- and HBV-related acute-on-chronic liver failure (ACLF) [13]. Additionally, serum levels of FT4 have been reported to be inversely associated with the severity of ACLF in patients with HAV and HBV infections, whereas patients with HAV and HBV infections without ACLF typically exhibit elevated TSH levels [12,13].

C-reactive protein (CRP), an inflammatory marker, offers crucial information about the immune response potential of the body [14]. Produced by hepatocytes in response to inflammation, CRP plays a role in processes such as apoptosis and phagocytosis [14]. Numerous meta-analyses have investigated the connection between CRP levels and HBV infection [9,14,15]. Some studies have suggested that increased serum CRP levels are correlated with the severity of HBV infection but not HAV infection and signify a high likelihood of liver damage, such as cirrhosis and fibrosis [2,15,16].

Typically, quantitative analysis techniques for specific antibodies, such as HAV and HBV antibodies, focus solely on assessing total antibody activity, with little emphasis on affinity maturation or antibody quality [2]. Thus, to achieve accurate and meaningful results, the binding affinity, protein content, and total antibody levels must be measured separately [2,6].

This study investigated a potential correlation between antibodies to HAV or HBV, inflammatory markers (CRP levels), and thyroid function indicators (TSH and FT4 levels). We hypothesized that individuals with HAV or HBV antibodies might have different CRP, TSH, and FT4 levels than those without antibodies. Additionally, we speculated that these correlations might vary according to demographic factors, such as age and the presence of antibodies to both HAV and HBV. Exploring these potential associations could provide valuable insights into the complex relationship between viral immunity, inflammation, and thyroid function, thereby shedding light on the underlying mechanisms of viral infections and their systemic effects.

This retrospective study aimed to evaluate and compare CRP, TSH, and FT4 levels in the serum of individuals who were vaccinated against HAV and HBV and compare them to unvaccinated healthy controls. The observed levels of CRP, TSH, and FT4 in individuals with HAV and HBV antibodies, irrespective of age and sex, indicate their potential utility as clinically valuable indicators of susceptibility to HAV and HBV infection. These findings contribute to our understanding and the management of patients with HAV and HBV infections, potentially leading to enhanced diagnostic strategies and improved patient outcomes.

## 2. Materials and Methods

### 2.1. Study Design and Participants

This retrospective study enrolled 31,026 individuals who visited Dankook University Hospital in Cheonan Province, South Korea, for medical checkups between 1 January 2022 and 31 December 2023. Individuals who did not undergo HAV and HBV antibody testing were excluded. The research protocol was reviewed and approved by the Clinical Research Review Committee of Dankook University (Institutional Review Board DKU, Certificate No. 2023-01-005-001). The requirement for informed consent was waived due to the retrospective study design. The study was conducted according to the principles of the Declaration of Helsinki.

**Figure 1:**
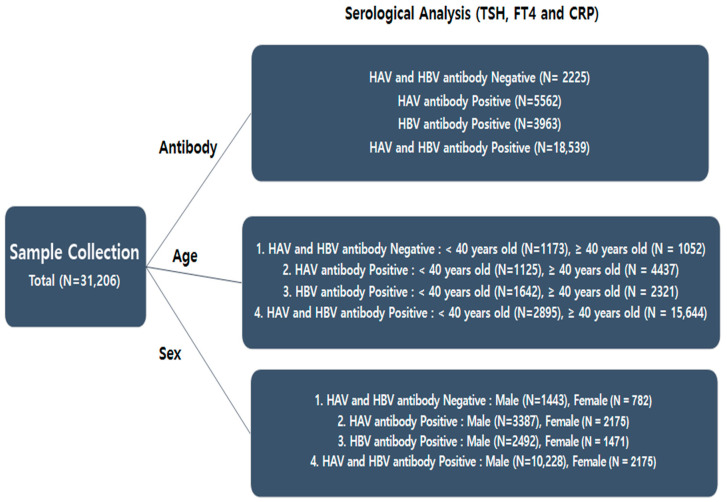
Study design and the number of participants enrolled in the study.

A subset of the participants (N = 30,289) underwent testing for HAV and HBV antibodies (Figure 1). The participants were stratified according to their HAV and HBV antibody status into antibody-positive and antibody-negative groups. To prevent potential cross-reactivity issues, we ensured there was no overlap of antibodies between these groups; for example, participants with HAV antibodies did not have HBV antibodies and vice versa. All participants were specifically chosen to be negative for HBV surface antigens (HBsAgs) to eliminate any potential interactions with the antigens. The participants were further categorized into distinct groups based on other clinical criteria, such as age, sex, and infection outcome. This systematic classification facilitated the examination of potential associations between HAV/HBV infection status and relevant variables. Participants with missing values for TSH, FT4, and CRP were excluded.

### 2.2. HAV and HBV Antibody Measurements

Serum samples were routinely tested for HAV and HBV antibodies at the laboratory of Dankook University Hospital in Cheonan. The following assays were used: Atellica IM Hepatitis A Total and Atellica IM Anti-Hepatitis B surface Antigen 2 assays by Siemens Healthcare Diagnostics, Tarrytown, NY, USA. The titer of HAV and HBV antibodies indicating positive infection were within the reference analytical range outlined by the respective assays, set at >20.0 mIU/mL for HAV antibodies and ≥10.0 mIU/mL for HBV antibodies. To ensure the quality of all measurements, quality controls were tested at least once during each work shift when analyzing samples. The quality control materials were used following the instructions provided. Additionally, the laboratory adhered to government regulations or accreditation requirements regarding the frequency of quality control.

### 2.3. CRP, TSH, and FT4 Measurements

The serum samples were subjected to routine analysis for inflammatory and thyroid markers using an electrochemiluminescence immunoassay. The concentrations of TSH, FT4, and CRP were within the reference ranges specified by Roche (Basel, Switzerland): 0.1–100.0 µIU/mL (TSH), 0.1–8.0 ng/dL (FT4), and 5–10 mg/L (CRP). These reference ranges are consistent with those outlined by the Korean Society of Laboratory Medicine: 0.6–4.84 µIU/mL (TSH), 0.97–1.67 ng/dL (FT4) [7], and <0.3 mg/dL (CRP) [17]. To maintain data quality, quality control measures were performed at least once during each work shift when analyzing the samples. The quality control materials were used in accordance with the provided instructions. Furthermore, the laboratory complied with governmental regulations or accreditation standards concerning the frequency of quality control.

### 2.4. Statistical Analysis

The continuous data, assumed to follow a normal distribution, were reported as means and standard deviations. One-way analysis of variance (ANOVA) with Bonferroni correction for multiple comparisons was performed to assess statistically significant differences between groups. All statistical analyses were performed using GraphPad Prism (Version 7.00.159, Dotmatics, Bishop’s Stortford, UK), with a predetermined level of statistical significance set at *p* < 0.05.

## 3. Results

### 3.1. Demographic and Clinical Characteristics

Among the 31,206 participants, 2225 did not have antibodies for HAV or HBV. For those remaining, 5562 had antibodies to HAV alone, 3963 had antibodies to HBV alone, and 18,539 had antibodies to both HAV and HBV (Table 1). No HAV or HBV antigens were detected in serum samples, as those with both antigens were excluded. The presence of antibodies for both HAV and HBV in 18,539 participants suggests past exposure or successful dual vaccination.

Compared with younger participants, participants aged 40 years and older were more likely to have both anti-HAV and anti-HBV antibodies (Table 2). Negative refers to participants who were negative for both HAV and HBV antibodies.

The HAV antibody-only group included 4820 male and 2957 female participants; the HBV antibody-only group included 3935 male and 2253 female participants; and the HAV and HBV antibody-positive group included 11,671 male and 9093 female participants (Table 3). Negative refers to participants who were negative for both HAV and HBV antibodies.

### 3.2. Inflammatory and Thyroid Parameters for Distinguishing between Participants with HAV and/or HBV Antibodies and Participants Negative for Both

As shown in Table 4, participants without antibodies for either HAV or HBV had CRP, TSH, and FT4 levels of 0.1268 ± 0.3036 mg/dL, 2.298 ± 1.790 µIU/mL, and 1.284 ± 1.285 ng/dL, respectively. Participants with anti-HAV antibodies had CRP, TSH, and FT4 levels of 0.1304 ± 0.2962 mg/dL, 2.310 ± 1.825 µIU/mL, and 1.276 ± 0.2411 ng/dL, respectively. Participants with anti-HBV antibodies had CRP, TSH, and FT4 levels of 0.1173 ± 0.3010 mg/dL, 2.226 ± 1.329 µIU/mL, and 1.272 ± 0.2104 ng/dL, respectively. Participants with both anti-HAV and anti-HBV antibodies had CRP, TSH, and FT4 levels of 0.1201 ± 0.3390 mg/dL, 2.319 ± 2.261 µIU/mL, and 1.272 ± 0.2104 ng/dL, respectively. To maintain statistical integrity, the data with non-Gaussian distributions are presented using the median along with the appropriate interquartile range (25–75%) in Supplementary Table S1.

After applying the Bonferroni correction for multiple comparisons, only the anti-HBV antibody-positive and anti-HAV and anti-HBV antibody-positive groups had significant differences in FT4 levels compared with the other groups (*p* < 0.01 for both) (Table 5).

### 3.3. CRP and Thyroid Hormone Levels According to Age and Anti-HAV and Anti-HBV Antibody Status

In the anti-HAV antibody-negative group, the CRP, TSH, and FT4 levels were 0.1348 ± 0.3434 mg/dL, 2.266 ± 1.631 µIU/mL, and 1.289 ± 0.2107 ng/dL, respectively, in participants aged under 40 years, and 0.1178 ± 0.2516 mg/dL, 2.333 ± 1.951 µIU/mL, and 1.277 ± 0.1901 ng/dL, respectively, in participants aged ≥ 40 years (Table 6).

In the anti-HAV antibody-positive group, the CRP, TSH, and FT4 levels were 0.1411 ± 0.3001 mg/dL, 2.296 ± 1.528 µIU/mL, and 1.297 ± 0.3033 ng/dL, respectively, in participants aged under 40 years, and 0.1277 ± 0.2952 mg/dL, 2.316 ± 1.764 µIU/mL, and 1.270 ± 0.2223 ng/dL, respectively, in participants aged ≥ 40 years (Table 6). After applying the Bonferroni correction, the FT4 levels differed significantly (*p* < 0.01) according to age (Supplementary Table S2).

In the anti-HBV antibody-negative group, the CRP, TSH, and FT4 levels were 0.1348 ± 0.3434 mg/dL, 2.266 ± 1.631 µIU/mL, and 1.289 ± 0.2107 ng/dL, respectively, in participants aged under 40 years, and 0.1178 ± 0.2516 mg/dL, 2.333 ± 1.951 µIU/mL, and 1.277 ± 0.1901 ng/dL, respectively, in participants aged ≥40 years. In the anti-HBV antibody-positive group, the CRP, TSH, and FT4 levels were 0.1279 ± 0.3264 mg/dL, 2.183 ± 1.142 µIU/mL, and 1.297 ± 0.2111 ng/dL, respectively, in participants aged under 40 years, and 0.1097 ± 0.2814 mg/dL, 2.257 ± 1.447 µIU/mL, and 1.276 ± 0.2011 ng/dL, respectively, in participants aged ≥ 40 years (Table 6). After applying the Bonferroni correction, the FT4 levels differed significantly (*p* < 0.01) according to age (Supplementary Table S3).

In the anti-HAV and anti-HBV antibody-positive group, the CRP, TSH, and FT4 levels were 0.1453 ± 0.5511 mg/dL, 2.281 ± 1.818 µIU/mL, and 1.273 ± 0.1923 ng/dL, respectively, in participants aged under 40 years, and 0.1154 ± 0.3664 mg/dL, 2.326 ± 2.333 µIU/mL, and 1.271 ± 0.2136 ng/dL, respectively, in participants aged ≥ 40 years (Table 6). After applying the Bonferroni correction, the CRP (*p* < 0.005) and FT4 (*p* < 0.05) levels differed significantly according to age (Supplementary Table S4).

Overall, there were no major variations in inflammatory and thyroid hormone levels among participants with or without anti-HAV and anti-HBV antibodies by age (Table 6). To uphold statistical integrity, data exhibiting non-Gaussian distributions are presented using the median and the corresponding interquartile range (25–75%) in Supplementary Table S5.

In evaluating the correlation between age and inflammatory and thyroid markers in relation to HAV and HBV antibody-positive status, the level of FT4 was significantly correlated with an HAV and HBV antibody-positive status individually, and an anti-HAV and anti-HBV antibody status combined (*p* = 0.004 for anti-HAV antibody status, *p* = 0.0049 for anti-HBV antibody status, and *p* = 0.0442 for both anti-HAV and anti-HBV antibodies). The CRP level was also significantly correlated with the anti-HAV and anti-HBV antibody status (*p* = 0.0397 for anti-HAV antibodies and *p* = 0.0336 for anti-HBV antibodies). Conversely, no significant correlation was observed between TSH levels and age according to anti-HAV and anti-HBV antibody status (Supplementary Table S6).

### 3.4. CRP and Thyroid Hormone Levels According to Sex and Anti-HAV and Anti-HBV Antibody Status

Male participants lacking antibodies for HAV and HBV had CRP, TSH, and FT4 levels of 0.1319 ± 0.2650 mg/dL, 2.180 ± 1.614 µIU/mL, and 1.301 ± 0.1974 ng/dL, respectively. In contrast, male participants with anti-HAV antibodies had levels of 0.1361 ± 0.3301 mg/dL, 2.207 ± 1.613 µIU/mL, and 1.291 ± 0.2266 ng/dL. Female participants without anti-HAV antibodies had CRP, TSH, and FT4 levels of 0.1173 ± 0.3641 mg/dL, 2.516 ± 2.059 µIU/mL, and 1.252 ± 0.2602 ng/dL, respectively, whereas those with anti-HAV antibodies had CRP, TSH, and FT4 levels of 0.1216 ± 0.2900 mg/dL, 2.476 ± 1.860 µIU/mL, and 1.251 ± 0.2602 ng/dL, respectively. Male participants with anti-HBV antibodies had CRP, TSH, and FT4 levels of 0.1230 ± 0.2742 mg/dL, 2.103 ± 1.231 µIU/mL, and 1.303 ± 0.2023 ng/dL, respectively, whereas female participants with anti-HBV antibodies had CRP, TSH, and FT4 levels of 0.1076 ± 0.3415 mg/dL, 2.434 ± 1.458 µIU/mL, and 1.254 ± 0.2074 ng/dL, respectively. Male participants with both anti-HAV and anti-HBV antibodies had CRP, TSH, and FT4 levels of 0.1301 ± 0.4495 mg/dL, 2.188 ± 1.768 µIU/mL, and 1.286 ± 0.2008 ng/dL, respectively, whereas female participants with both anti-HAV and anti-HBV antibodies had CRP, TSH, and FT4 levels of 0.1078 ± 0.3259 mg/dL, 2.516 ± 2.059 µIU/mL, and 1.252 ± 0.2044 ng/dL, respectively (Table 7).

In participants without anti-HAV or anti-HBV antibodies, thyroid hormone levels (*p* < 0.001) were significantly lower in female participants than in male participants. The measured values were below the reference ranges. After correcting for multiple comparisons using the Bonferroni test, sex-related differences in TSH and FT4 levels were significant, whereas differences related to HAV or HBV antibody status were not (Supplementary Tables S7–S9). The absence of significant differences in anti-HBV antibody status was consistent with the findings for anti-HAV antibodies. In participants with both anti-HAV and anti-HBV antibodies, only the CRP level differed significantly between male and female participants. To ensure statistical integrity, data with non-Gaussian distributions are reported as the median along with the interquartile range (25–75%) in Supplementary Table S10.

## 4. Discussion

This study measured CRP, TSH, and FT4 levels in individuals with and without anti-HAV and anti-HBV antibodies and found that both groups had markers within the reference ranges. We explored the correlations between anti-HAV and anti-HBV antibodies, CRP levels as a marker of inflammation, and thyroid hormone levels to gain insight into the broader physiological effects of viral infections. By considering demographic factors, such as age, sex, and the presence of antibodies, this study sought to identify patterns that could inform personalized patient management and lead to targeted strategies for treating viral infections.

Our study provides a comprehensive analysis of HAV and HBV immunity, examining both acute (HAV) and potentially chronic (HBV) infections. This approach helps identify differences and similarities in immune responses and offers insights into long-term immunity as a result of vaccination. Given the higher prevalence of these viruses in Korea compared with other hepatitis viruses, such as hepatitis C virus (HCV), hepatitis D virus, and hepatitis E virus, addressing HAV and HBV simultaneously aids in developing better prevention and treatment strategies. This research lays the foundation for future studies on concurrent or sequential infections and their effects on immune responses and disease outcomes in Korea.

This study found that the majority of participants had antibodies for both HAV and HBV. This underscores the success of vaccination efforts and the importance of dual immunity in public health strategies, emphasizing the need for widespread vaccination coverage to enhance protection [1,2,3].

The higher prevalence of anti-HAV and anti-HBV antibody positivity combined, compared with antibodies to HAV or HBV alone among older adults, indicates age-related differences in exposure or susceptibility. These findings can inform targeted public health interventions to reduce hepatitis A and B infections in various demographics. Further research is necessary to identify the factors contributing to these disparities.

The CRP and TSH levels did not differ significantly according to anti-HAV and anti-HBV antibody status, but FT4 levels differed. Our study identified higher average CRP levels in participants with anti-HBV antibodies in contrast to prior research that reported elevated mean CRP levels in participants with the HBV antigen [14,16,17]. Limited information is available on the relationship between inflammatory function and immunity to viral hepatitis. Previous studies have shown that FT4 levels can decrease and TSH levels increase in cirrhosis, with no changes in hepatitis B [18,19]. Further research is needed to understand why FT4 levels are associated with anti-HAV and anti-HBV antibodies, whereas CRP and TSH levels are not.

In the anti-HAV and anti-HBV antibody-negative group, no significant differences were observed in CRP, TSH, and FT4 levels according to age. Similarly, minimal and non-significant variations were seen in antibody-positive groups according to age. Previous studies have shown that TSH levels increase with age in healthy older adults [20,21,22], and CRP levels are higher in individuals with HBV infection across all ages [16,23]. However, research on the relationship between immunity to viral hepatitis and thyroid or inflammatory function across different age groups is limited. In participants with anti-HAV antibodies, the Bonferroni test revealed significant differences in FT4 levels in patients under 40 years compared with those aged 40 years or older. Similarly, in participants with anti-HBV antibodies, FT4 levels varied significantly with age, whereas CRP and TSH levels did not. This consistent pattern across anti-HAV and anti-HBV antibody groups underscores the reliability of FT4 as an indicator for evaluating the influence of viral antibodies on thyroid function.

The TSH and FT4 levels differed according to sex, regardless of the anti-HAV and anti-HBV antibody status, suggesting that sex hormones may interact with viral immunity to affect thyroid function differently in males and females [22]. Although CRP levels did not vary significantly according to anti-HAV and anti-HBV antibody status, TSH and FT4 levels varied, indicating that sex is an effect modifier. Previous studies have shown declining TSH and FT4 in aging women [18,22] and stable CRP levels across sexes [16,23]. The significant variations in thyroid hormones and CRP levels in participants with both anti-HAV and anti-HBV antibodies suggest that dual immunity may complicate the immune environment. These findings underscore the importance of considering sex-specific responses and multiple viral antibodies in evaluating thyroid function and inflammatory markers. Further research is needed to explore the mechanisms behind these differences and their clinical implications.

Our study has some limitations, including missing data on additional thyroid biomarkers, including total T3 and free T3, which are important for assessing thyroid function. Despite this, after confirming anti-HAV and anti-HBV antibody status, TSH and FT4 levels showed potential for tracking thyroid function [11]. Although direct causal relationships between inflammatory biomarkers and anti-HAV and anti-HBV antibody levels are difficult to establish, CRP monitoring post-infection is valuable for tracking inflammation [14,15,23,24]. Future research should include additional cytokines, such as IL-6, IL-8, D-dimer, and TNF-alpha, to enhance predictive capabilities. This study’s retrospective nature and lack of clinical data on vaccination history and use of thyroid medications underscore the need for further investigation into these areas and the effect of physiological differences by sex and age [8,25,26]. This retrospective study did not have complete clinical data, such as signs, symptoms, test results, and treatment information. To address these limitations, we used measures such as data verification, seeking additional materials, and cross-checking information from multiple sources. We have also clearly outlined the limitations to account for potential biases.

Our research, focusing on individuals with confirmed antibodies to HAV and HBV, could not distinguish between naturally acquired and vaccine-induced immunity due to data constraints. This highlights the need for future studies to investigate how different methods of acquiring immunity affect metabolic activity, providing deeper insights into immune responses and their physiological effects.

Given the notable metabolic changes observed in individuals with anti-HAV and anti-HBV antibodies, it is crucial to investigate the relationship between anti-HAV and anti-HBV antibody levels and hormone levels to determine if heightened immunity affects metabolic biomarkers. Previous research reported that individuals with anti-HCV antibodies have lower mean CRP levels than negative anti-HCV individuals [27,28,29], and HCV infection can lead to thyroid dysfunction [30]. Thus, examining the metabolic effects of HCV immunity is essential.

## 5. Conclusions

This study analyzed CRP, TSH, and FT4 levels in individuals with and without anti-HAV and anti-HBV antibodies. Participants without antibodies and those with either anti-HAV or anti-HBV antibodies had normal levels of these markers. Stratifying by age and sex revealed differences in antibody prevalence by age and sex, suggesting disparities in exposure or susceptibility. Whereas CRP and TSH levels were stable, FT4 levels varied, particularly among those with anti-HAV antibodies or both anti-HAV and anti-HBV antibodies, indicating possible associations with thyroid function, especially in younger participants. Significant variations in thyroid hormones by sex and antibody status suggest complex interactions between viral immunity and thyroid function. Despite limitations, such as missing data on additional thyroid biomarkers and clinical details, this study highlights the utility of monitoring CRP in HAV and HBV infection. Future research should examine additional cytokines and differentiate between natural and vaccine-induced immunity to better understand the effects of these infections on inflammatory and thyroid hormone levels.

## Figures and Tables

**Table 1:** HAV and HBV antibody status of the participants.

Antibody Status	Number	%
HAV and HAV negative	2225	7.35
HAV positive, HBV negative	5562	18.36
HAV negative, HBV positive	3963	13.08
HAV and HAV positive	18,539	61.21

HAV, hepatitis A virus; HBV, hepatitis B virus.

**Table 2:** HAV and HBV antibody status of the participants according to age.

Antibody Status		Age < 40 Years	Age ≥ 40 Years
Number of participants with HAV Ab	Total tested	2298	5489
	Negative	1173	1052
	Positive	1125	4437
Number of participants with HBV Ab	Total tested	2815	3373
	Negative	1173	1052
	Positive	1642	2321
Number of participants with HAV and HBV Ab	Total tested	4068	16,696
	Negative	1173	1052
	Positive	2895	15,644

Ab, antibody; HAV, hepatitis A virus; and HBV, hepatitis B virus.

**Table 3:** HAV and HBV antibody status of the participants according to sex.

Antibody Status		Male	Female
Number of HAV Ab-positive participants	Total tested	4820	2957
	Negative	1443	782
	Positive	3387	2175
Number of HBV Ab-positive participants	Total tested	3935	2253
	Negative	1443	782
	Positive	2492	1471
Number of HAV and HBV Ab-positive participants	Total tested	11,671	9093
	Negative	1443	782
	Positive	10,228	8311

Ab, antibody; HAV, hepatitis A virus; and HBV, hepatitis B virus.

**Table 4:** Inflammatory (CRP) and thyroid parameters (TSH and FT4) according to HAV and HBV antibody status.

	Antibody Status			
	Anti-HAV and Anti-HBV Ab Negative (N = 2225)	Only Anti-HAV Ab Positive (N = 5562)	Only Anti-HBV Ab Positive (N = 3963)	Anti-HAV and Anti-HBV Ab Positive (N = 18,539)
CRP (mg/dL)	0.1268 ± 0.3036	0.1304 ± 0.2962	0.1173 ± 0.3010	0.1201 ± 0.3390
TSH (µIU/mL)	2.298 ± 1.790	2.310 ± 1.825 ^†^	2226 ± 1.329	2.319 ± 2.261
FT4 (ng/dL)	1.284 ± 1.285	1.276 ± 0.2411	1.285 ± 0.2055	1.272 ± 0.2104

^†^ N = 3886. Values are reported as means and standard deviations. Ab, antibody; CRP, C-reactive protein; FT4, free thyroxine; HAV, hepatitis A virus; HBV, hepatitis B virus; and TSH, thyroid-stimulating hormone. *p* = 0.2068 for CRP, *p* = 0.0801 for TSH, and *p* = 0.0012 for FT4.

**Table 5:** Statistical analysis of inflammatory and thyroid parameters according to antibody status using the Bonferroni correction for multiple comparisons.

Parameter	Comparison	Mean Diff.	*t*	*p* < 0.05?	Summary	95% CI of Diff.
CRP (mg/dL)	Negative vs. anti-HAV Ab +	−0.003644	0.3996	No	NS	−0.02771 to 0.02042
	Negative vs. anti-HBV Ab +	0.009514	0.9878	No	NS	−0.01590 to 0.03493
	Negative vs. anti-HAV and anti-HBV Ab +	0.006710	0.8226	No	NS	−0.01481 to 0.02823
	Anti-HAVAb + vs. anti-HBV Ab +	0.01316	1.741	No	NS	−0.006783 to 0.03310
	Anti-HAV Ab + vs. anti-HAV and anti-HBV Ab +	0.01035	1.863	No	NS	−0.004312 to 0.02502
	Anti-HBV Ab + vs. anti-HAV and anti-HBV Ab +	−0.002804	0.4407	No	NS	−0.01959 to 0.01398
TSH (µIU/mL)	Negative vs. anti-HAV Ab +	−0.01164	0.2122	No	NS	−0.1564 to 0.1331
	Negative vs. anti-HBV Ab +	0.07214	1.320	No	NS	−0.07207 to 0.2164
	Negative vs. anti-HAV and anti-HBV Ab +	−0.02136	0.4613	No	NS	−0.1435 to 0.1008
	Anti-HAVAb + vs. anti-HBV Ab +	0.08378	1.799	No	NS	−0.03912 to 0.2067
	Anti-HAV Ab + vs. anti-HAV and anti-HBV Ab +	−0.009714	0.2669	No	NS	−0.1058 to 0.08633
	Anti-HBV Ab + vs. anti-HAV and anti-HBV Ab +	−0.09350	2.589	No	NS	−0.1888 to 0.001776
FT4 (ng/dL)	Negative vs. anti-HAV Ab +	0.008080	1.497	No	NS	−0.006158 to 0.02232
	Negative vs. anti-HBV Ab +	−0.001072	0.1881	No	NS	−0.01611 to 0.01396
	Negative vs. anti-HAV and anti-HBV Ab +	0.01185	2.456	No	NS	−0.0008803 to 0.02459
	Anti-HAVAb + vs. anti-HBV Ab +	−0.009152	2.047	No	NS	−0.02095 to 0.002647
	Anti-HAV Ab + vs. anti-HAV and anti-HBV Ab +	0.003774	1.148	No	NS	−0.004904 to 0.01245
	Anti-HBV Ab + vs. anti-HAV and anti-HBV Ab +	0.01293	3.433	Yes	**	0.002993 to 0.02286

Ab, antibody; CI, confidence interval; CRP, C-reactive protein; diff., difference; FT4, free thyroxine; HAV, hepatitis A virus; HBV, hepatitis B virus; NS, not significant; and TSH, thyroid-stimulating hormone. ** *p* < 0.01.

**Table 6:** Inflammatory (CRP) and thyroid parameters (TSH and FT4) according to age and HAV, HBV, and both HAV and HBV antibody status.

	Antibody Negative		Antibody Positive	
	Age < 40 Years	Age ≥ 40 Years	Age < 40 Years	Age ≥ 40 Years
Anti-HAV Ab	(N = 1173)	(N = 1052)	(N = 1125)	(N = 4437)
CRP (mg/dL)	0.1348 ± 0.3434	0.1178 ± 0.2516	0.1411 ± 0.3001	0.1277 ± 0.2952
TSH (µIU/mL)	2.266 ± 1.631	2.333 ± 1.951	2.296 ± 1.528	2.316 ± 1764
FT4 (ng/dL)	1.289 ± 0.2107	1.277 ± 0.1901	1.297 ± 0.3033	1.270 ± 0.2223
Anti-HBV Ab	(N = 1173)	(N = 1052)	(N = 1642)	(N = 2321)
CRP (mg/dL)	0.1348 ± 0.3434)	0.1178 ± 0.2516	0.1279 ± 0.3264	0.1097 ± 0.2814
TSH (µIU/mL)	2.266 ± 1.631	2.333 ± 1.951	2.181 ± 1.142	2.257 ± 1.447
FT4 (ng/dL)	1.289 ± 0.2107	1.277 ± 0.1901	1.297 ± 0.2111	1.276 ± 0.2011
Anti-HAV and anti-HBV Ab	(N = 1173)	(N = 1052)	(N = 2895)	(N = 15,644)
CRP (mg/dL)	0.1348 ± 0.3434	0.1178 ± 0.2516)	0.1433 ± 0.5512	0.1154 ± 0.3637
TSH (µIU/mL)	2.266 ± 1.631	2.333 ± 1.951	2.281 ± 1.818	2.326 ± 2.333
FT4 (ng/dL)	1.289 ± 0.2107	1.277 ± 0.1901	1.273 ± 0.1923	1.271 ± 0.2136

Ab, antibody; CRP, C-reactive protein; FT4, free thyroxine; HAV, hepatitis A virus; HBV, hepatitis B virus; and TSH, thyroid-stimulating hormone.

**Table 7:** Inflammatory (CRP) and thyroid parameters (TSH and FT4) according to sex and HAV, HBV, and HAV and HBV antibody status.

	Male		Female	
	Negative	Positive	Negative	Positive
Anti-HAV Ab	(N = 1443)	(N = 3387)	(N = 782)	(N = 2175)
CRP (mg/dL)	0.1319 ± 0.2650	0.1361 ± 0.3001	0.1173 ± 0.3641	0.1216 ± 0.2990
TSH (µIU/mL)	2.280 ± 1.614	2.207 ± 1.613	2.516 ± 2.059	2.476 ± 1.860
FT4 (ng/dL)	1.301 ± 0.1974	1.291 ± 0.2266	1.252 ± 0.2044	1.251 ± 0.2602
Anti-HBV Ab	(N = 1443)	(N = 2492)	(N = 782)	(N = 1471)
CRP (mg/dL)	0.1319 ± 0.2650	0.1230 ± 0.2742	0.1173 ± 0.3641	0.1076 ± 0.3415
TSH (µIU/mL)	2.180 ± 1.614	2.103 ± 1.231	2516 ± 2.059	2.434 ± 1.458
FT4 (ng/dL)	1.301 ± 0.1974	1.303 ± 0.2303	1.252 ± 0.2044	1.254 ± 0.2074
Anti-HAV and anti-HBV Ab	(N = 1443)	(N = 10,228)	(N = 782)	(N = 8311)
CRP (mg/dL)	0.1319 ± 0.2650	0.1301 ± 0.4495	0.1173 ± 0.3641	0.1078 ± 0.3259
TSH (µIU/mL)	2.180 ± 1.614	2.333 ± 1.951	2.516 ± 2.059	2.481 ± 2.740
FT4 (ng/dL)	1.301 ± 0.1974	1.286 ± 0.2008	1.252 ± 0.2044	1.254 ± 0.2205

Ab, antibody; CRP, C-reactive protein; FT4, free thyroxine; HAV, hepatitis A virus; HBV, hepatitis B virus; and TSH, thyroid-stimulating hormone.

## Data Availability

The datasets utilized and examined in this study can be obtained from the corresponding author upon a request that is deemed reasonable.

## Supplementary Materials

The following supporting information can be downloaded at https://www.mdpi.com/article/10.3390/v16081329/s1. Table S1. The median and interquartile range (25%–75%) of inflammatory (CRP) and thyroid parameters (TSH and FT4) according to HAV and HBV antibody status; Table S2. Statistical analysis of inflammatory (CRP) and thyroid parameters (TSH and FT4) based on age and HAV antibody status with the Bonferroni correction for multiple comparisons; Table S3. Statistical analysis of inflammatory (CRP) and thyroid parameters (TSH and FT4) based on age and HBV antibody status with the Bonferroni correction for multiple comparisons; Table S4. Statistical analysis of inflammatory (CRP) and thyroid parameters (TSH and FT4) based on age and both HAV and HBV antibody status with the Bonferroni correction for multiple comparisons; Table S5. The median and interquartile range (25%–75%) of inflammatory (CRP) and thyroid parameters (TSH and FT4) according to age and HAV, HBV and both HAV and HBV antibody status; Table S6. Correlation and linear regression analysis of inflammatory marker (CRP) and thyroid parameters (TSH and FT4) in relation to age and HAV and HBV antibody status; Table S7. Statistical analysis of inflammatory (CRP) and thyroid parameters (TSH and FT4) based on sex and HAV antibody status with the Bonferroni correction for multiple comparisons; Table S8. Statistical analysis of inflammatory (CRP) and thyroid parameters (TSH and FT4) based on sex and HBV antibody status with the Bonferroni correction for multiple comparisons; Table S9. Statistical analysis of inflammatory (CRP) and thyroid parameters (TSH and FT4) based on sex and both HAV and HBV antibody status with the Bonferroni correction for multiple comparisons; Table S10. The median and interquartile range (25%–75%) of inflammatory (CRP) and thyroid parameters (TSH and FT4) based on sex and both HAV and HBV antibody status.
