# Supplementary material for: Analysis of Inflammatory and Thyroid Hormone Levels Based on Hepatitis A and B Virus Immunity Status: Age and Sex Stratification
Source: Viruses. 2024 Aug 20;16(8):1329. doi: 10.3390/v16081329 (PMC11358917; doi:10.3390/v16081329)
Supplement: Supplementary file 1 [file viruses-16-01329-s001.zip › viruses-3071004-supplementary.pdf]

Supplementary Table S1. The median and interquartile range (25%-75%) of inflammatory (CRP) and thyroid parameters (TSH and FT4) according to HAV and HBV antibody status.

		Antibody status			
		**Anti-HAV and anti-HBV Ab negative (N = 2225)**	**Only anti-HAV Ab positive (N = 5562)**	**Only anti-HBV Ab positive (N = 3963)**	**Anti-HAV and anti-HBV Ab positive (N = 18,539)**
**CRP**	25% Percentile	0.0200	0.0300	0.0200	0.0200
	Median	0.0500	0.0500	0.0500	0.0500
	75% Percentile	0.1200	0.1100	0.1100	0.1000
**TSH**	25% Percentile	1.363	1.360[†]	1.330	1.350
	Median	2.140	2.220[†]	2.125	2.160
	75% Percentile	2.830	2.880[†]	2.820	2.850
**FT4**	25% Percentile	1.150	1.140	1.160	1.140
	Median	1.260	1.250	1.270	1.250
	75% Percentile	1.380	1.380	1.390	1.380

[†] N = 3,886. Values are reported as means and standard deviations. Ab, antibody; CRP, C-reactive protein; FT4, free thyroxine; HAV, hepatitis A virus; HBV, hepatitis B virus; TSH, thyroid-stimulating hormone. $p = 0.2068$ for CRP, $p = 0.0801$ for TSH, and $p = 0.0012$ for FT4.

Supplementary Table S2. Statistical analysis of inflammatory (CRP) and thyroid parameters (TSH and FT4) based on age and HAV antibody status with the Bonferroni correction for multiple comparisons.

Parameter	HAV Ab and age comparison	Mean diff.	t	*P* < 0.05?	Summary	95% CI of diff.
CRP (mg/dL)	Negative (< 40 years) vs negative (≥ 40 years)	0.01696	1.339	No	**NS**	−0.01647 to 0.05039
	Negative (< 40 years) vs positive (< 40 years)	−0.006343	0.5095	No	**NS**	−0.03920 to 0.02651
	Negative (< 40 years) vs positive (≥ 40 years)	0.007095	0.7244	No	**NS**	−0.01875 to 0.03294
	Negative (≥ 40 years) vs positive (< 40 years)	−0.02331	1.822	No	**NS**	−0.05707 to 0.01046
	Negative (≥ 40 years) vs positive (≥ 40 years)	−0.009869	0.9647	No	**NS**	−0.03687 to 0.01713
	Positive (< 40 years) vs positive (≥ 40 years)	0.01344	1.349	No	**NS**	−0.01284 to 0.03972
TSH (µIU/mL)	Negative (< 40 years) vs negative (≥ 40 years)	−0.06650	0.9003	No	**NS**	−0.2614 to 0.1284
	Negative (< 40 years) vs positive (< 40 years)	−0.02958	0.4076	No	**NS**	−0.2211 to 0.1620
	Negative (< 40 years) vs positive (≥ 40 years)	−0.04972	0.8706	No	**NS**	−0.2004 to 0.1010
	Negative (≥ 40 years) vs positive (< 40 years)	0.03691	0.4948	No	**NS**	−0.1599 to 0.2338
	Negative (≥ 40 years) vs positive (≥ 40 years)	0.01678	0.2813	No	**NS**	−0.1406 to 0.1742
	Positive (< 40 years) vs positive (≥ 40 years)	−0.02013	0.3467	No	**NS**	−0.1734 to 0.1331
FT4 (ng/dL)	Negative (< 40 years) vs negative (≥ 40 years)	0.01186	1.213	No	**NS**	−0.01394 to 0.03766
	Negative (< 40 years) vs positive (< 40 years)	−0.007744	0.8060	No	**NS**	−0.03310 to 0.01761
	Negative (< 40 years) vs positive (≥ 40 years)	0.01912	2.530	No	**NS**	−0.0008266 to 0.03907
	Negative (≥ 40 years) vs positive (< 40 years)	−0.01960	1.985	No	**NS**	−0.04566 to 0.006456
	Negative (≥ 40 years) vs positive (≥ 40 years)	0.007263	0.9199	No	**NS**	−0.01357 to 0.02810
	Positive (< 40 years) vs positive (≥ 40 years)	0.02687	3.496	Yes	**	0.006584 to 0.04715

Ab, antibody; CI, confidence interval; CRP, C-reactive protein; diff., difference; FT4, free thyroxine; HAV, hepatitis A virus; NS, not significant; TSH, thyroid-stimulating hormone. ** $p < 0.01$.

Supplementary Table S3. Statistical analysis of inflammatory (CRP) and thyroid parameters (TSH and FT4) based on age and HBV antibody status with the Bonferroni correction for multiple comparisons.

Parameter	HBV Ab and age comparison	Mean diff.	t	*P* < 0.05?	Summary	95% CI of diff.
CRP (mg/dL)	Negative (< 40 years) vs negative (≥ 40 years)	0.01696	1.324	No	**NS**	−0.01686 to 0.05079
	Negative (< 40 years) vs positive (< 40 years)	0.006906	0.5985	No	**NS**	−0.02355 to 0.03736
	Negative (< 40 years) vs positive (≥ 40 years)	0.02505	2.317	No	**NS**	−0.003481 to 0.05359
	Negative (≥ 40 years) vs positive (< 40 years)	−0.01006	0.8438	No	**NS**	−0.04151 to 0.02140
	Negative (≥ 40 years) vs positive (≥ 40 years)	0.008090	0.7212	No	**NS**	−0.02152 to 0.03770
	Positive (< 40 years) vs positive (≥ 40 years)	0.01815	1.865	No	**NS**	−0.007538 to 0.04383
TSH (μIU/mL)	Negative (< 40 years) vs negative (≥ 40 years)	−0.06650	1.036	No	**NS**	−0.2358 to 0.1028
	Negative (< 40 years) vs positive (< 40 years)	0.08501	1.472	No	**NS**	−0.06744 to 0.2375
	Negative (< 40 years) vs positive (≥ 40 years)	0.009357	0.1729	No	**NS**	−0.1335 to 0.1522
	Negative (≥ 40 years) vs positive (< 40 years)	0.1515	2.539	No	**NS**	−0.005977 to 0.3090
	Negative (≥ 40 years) vs positive (≥ 40 years)	0.07585	1.351	No	**NS**	−0.07236 to 0.2241
	Positive (< 40 years) vs positive (≥ 40 years)	−0.07565	1.553	No	**NS**	−0.2042 to 0.05294
FT4 (ng/dL)	Negative (< 40 years) vs negative (≥ 40 years)	0.01186	1.370	No	**NS**	−0.01098 to 0.03470
	Negative (< 40 years) vs positive (< 40 years)	−0.008007	1.028	No	**NS**	−0.02857 to 0.01256
	Negative (< 40 years) vs positive (≥ 40 years)	0.01341	1.836	No	**NS**	−0.005864 to 0.03268
	Negative (≥ 40 years) vs positive (< 40 years)	−0.01987	2.468	No	**NS**	−0.04111 to 0.001379
	Negative (≥ 40 years) vs positive (≥ 40 years)	0.001550	0.2045	No	**NS**	−0.01844 to 0.02154
	Positive (< 40 years) vs positive (≥ 40 years)	0.02142	3.258	Yes	**	0.004068 to 0.03876

Ab, antibody; CI, confidence interval; CRP, C-reactive protein; diff., difference; FT4, free thyroxine; HBV, hepatitis B virus; NS, not significant; TSH, thyroid-stimulating hormone. ** $p < 0.01$.

Supplementary Table S4. Statistical analysis of inflammatory (CRP) and thyroid parameters (TSH and FT4) based on age and both HAV and HBV antibody status with the Bonferroni correction for multiple comparisons.

Parameter	HAV+HBV Ab and age comparison	Mean diff.	t	*P* < 0.05?	Summary	95% CI of diff.
CRP (mg/dL)	Negative (< 40 years) vs negative (≥ 40 years)	0.01696	1.025	No	**NS**	−0.02671 to 0.06063
	Negative (< 40 years) vs positive (< 40 years)	−0.01047	0.7762	No	**NS**	−0.04607 to 0.02512
	Negative (< 40 years) vs positive (≥ 40 years)	0.01939	1.644	No	**NS**	−0.01174 to 0.05053
	Negative (≥ 40 years) vs positive (< 40 years)	−0.02743	1.955	No	**NS**	−0.06446 to 0.009588
	Negative (≥ 40 years) vs positive (≥ 40 years)	0.002431	0.1958	No	**NS**	−0.03033 to 0.03519
	Positive (< 40 years) vs positive (≥ 40 years)	0.02987	3.787	Yes	***	0.009058 to 0.05067
TSH (μIU/mL)	Negative (< 40 years) vs negative (≥ 40 years)	−0.06650	0.7070	No	**NS**	−0.3147 to 0.1817
	Negative (< 40 years) vs positive (< 40 years)	−0.01476	0.1925	No	**NS**	−0.2170 to 0.1875
	Negative (< 40 years) vs positive (≥ 40 years)	−0.05983	0.8924	No	**NS**	−0.2368 to 0.1171
	Negative (≥ 40 years) vs positive (< 40 years)	0.05174	0.6489	No	**NS**	−0.1586 to 0.2621
	Negative (≥ 40 years) vs positive (≥ 40 years)	0.006662	0.09443	No	**NS**	−0.1795 to 0.1928
	Positive (< 40 years) vs positive (≥ 40 years)	−0.04508	1.006	No	**NS**	−0.1633 to 0.07316
FT4 (ng/dL)	Negative (< 40 years) vs negative (≥ 40 years)	0.01186	1.333	No	**NS**	−0.01161 to 0.03533
	Negative (< 40 years) vs positive (< 40 years)	0.01573	2.169	No	**NS**	−0.003403 to 0.03486
	Negative (< 40 years) vs positive (≥ 40 years)	0.01778	2.804	Yes	*	0.001051 to 0.03451
	Negative (≥ 40 years) vs positive (< 40 years)	0.003869	0.5130	No	**NS**	−0.01603 to 0.02377
	Negative (≥ 40 years) vs positive (≥ 40 years)	0.005924	0.8879	No	**NS**	−0.01168 to 0.02353
	Positive (< 40 years) vs positive (≥ 40 years)	0.002056	0.4850	No	**NS**	−0.009127 to 0.01324

Ab, antibody; CI, confidence interval; CRP, C-reactive protein; diff., difference; FT4, free thyroxine; HAV, hepatitis A virus; HBV, hepatitis B virus; NS, not significant; TSH, thyroid-stimulating hormone. * $p < 0.05$; *** $p < 0.001$.

Supplementary Table S5. The median and interquartile range (25%-75%) of inflammatory (CRP) and thyroid parameters (TSH and FT4) according to age and HAV, HBV and both HAV and HBV antibody status.

		Antibody negative		Antibody positive	
		Age < 40 years	Age ≥ 40 years	Age < 40 years	Age ≥ 40 years
**Anti-HAV Ab**		(N = 1173)	(N = 1052)	(N = 1125)	(N = 4437)
**CRP**	25% Percentile	0.0200	0.0300	0.0200	0.0300
	Median	0.0500	0.0600	0.0500	0.0600
	75% Percentile	0.1205	0.1200	0.1300	0.1150
**TSH**	25% Percentile	1.340	1.383	1.365	1.340
	Median	2.110	2.190	2.270	2.180
	75% Percentile	2.825	2.840	2.880	2.870
**FT4**	25% Percentile	1.160	1.150	1.170	1.140
	Median	1.270	1.250	1.270	1.250
	75% Percentile	1.390	1.380	1.390	1.370
**Anti-HBV Ab**		(N = 1173)	(N = 1052)	(N = 1642)	(N = 2321)
**CRP**	25% Percentile	0.0200	0.0300	0.0200	0.0200
	Median	0.0500	0.0600	0.0500	0.0500
	75% Percentile	0.1205	0.1200	0.1200	0.1100
**TSH**	25% Percentile	1.340	1.383	1.320	1.340
	Median	2.110	2.190	2.070	2.160
	75% Percentile	2.825	2.840	2.790	2.840
**FT4**	25% Percentile	1.160	1.150	1.170	1.160
	Median	1.270	1.250	1.275	1.260
	75% Percentile	1.390	1.380	1.403	1.370
**Anti-HAV and anti-HBV Ab**		(N = 1173)	(N = 1052)	(N = 2895)	(N = 15,644)
**CRP**	25% Percentile	0.0200	0.0300	0.0200	0.0200
	Median	0.0500	0.0600	0.0500	0.0500
	75% Percentile	0.1205	0.1200	0.1200	0.1000
**TSH**	25% Percentile	1.340	1.383	1.363	1.350
	Median	2.110	2.190	2.140	2.170
	75% Percentile	2.825	2.840	2.818	2.860

	25% Percentile	1.160	1.150	1.150	1.140
**FT4**	Median	1.270	1.250	1.260	1.250
	75% Percentile	1.390	1.380	1.380	1.370

Ab, antibody; CRP, C-reactive protein; FT4, free thyroxine; HAV, hepatitis A virus; HBV, hepatitis B virus; TSH, thyroid-stimulating hormone.

Supplementary Table S6. Correlation and linear regression analysis of inflammatory marker (CRP) and thyroid parameters (TSH and FT4) in relation to age and HAV and HBV antibody status.

HAV and HBV Ab status		CRP	TSH	FT4
**HAV antibody-positive**	Number of Pairs	5,562	5,560	5,561
	R	0.02758	0.02397	−0.04735
	P-value	0.0397	0.0739	0.004
	Significance	*	NS	***
	Linear regression slope	0.0006740 to 0.0003276	0.003398 to 0.001901	−0.0009345 to 0.0002658
**HBV antibody-positive**	Number of Pairs	3,963	3,962	3,963
	R	−0.03376	0.002082	−0.04472
	P-value	0.0336	0.8958	0.0049
	Significance	*	NS	**
	Linear regression slope	−0.001544 to 0.0007262	0.0004205 to 0.003209	−0.001397 to 0.0004957
**HAV and HBV antibody-positive**	Number of Pairs	18,540	18,540	18,540
	R	0.008427	0.006318	−0.01478
	P-value	0.2512	0.3896	0.0442
	Significance	NS	NS	*
	Linear regression slope	0.0003278 to 0.0002857	0.001392 to 0.001618	−0.0003032 to 0.0001507

Ab, antibody; CRP, C-reactive protein; FT4, free thyroxine; HAV, hepatitis A virus; HBV, hepatitis B virus; NS, not significant; TSH, thyroid-stimulating hormone. * $p < 0.05$; ** $p < 0.01$; *** $p < 0.001$.

Supplementary Table S7. Statistical analysis of inflammatory (CRP) and thyroid parameters (TSH and FT4) based on sex and HAV antibody status with the Bonferroni correction for multiple comparisons.

Parameter	HAV Ab and sex comparison	Mean diff.	t	*P* < 0.05?	Summary	95% CI of diff.
CRP (mg/dL)	Negative M vs positive M	−0.004194	0.4472	No	**NS**	−0.02894 to 0.02055
	Negative M vs negative F	0.01457	1.100	No	**NS**	−0.02039 to 0.04952
	Negative M vs positive F	0.01030	1.017	No	**NS**	−0.01642 to 0.03703
	Positive M vs negative F	0.01876	1.585	No	**NS**	−0.01247 to 0.04999
	Positive M vs positive F	0.01450	1.769	No	**NS**	−0.007133 to 0.03613
	Negative F vs positive F	−0.004262	0.3426	No	**NS**	−0.03709 to 0.02856
TSH (µIU/mL)	Negative M vs positive M	−0.02675	0.4908	No	**NS**	−0.1706 to 0.1171
	Negative M vs negative F	−0.3358	4.362	Yes	***	−0.5390 to -0.1327
	Negative M vs positive F	−0.2965	5.036	Yes	***	−0.4518 to -0.1411
	Positive M vs negative F	−0.3091	4.494	Yes	***	−0.4906 to -0.1276
	Positive M vs positive F	−0.2697	5.661	Yes	***	−0.3954 to -0.1440
	Negative F vs positive F	0.03939	0.5449	No	**NS**	−0.1514 to 0.2302
FT4 (ng/dL)	Negative M vs positive M	0.009367	1.298	No	**NS**	−0.009670 to 0.02840
	Negative M vs negative F	0.04906	4.815	Yes	***	0.02217 to 0.07595
	Negative M vs positive F	0.05017	6.439	Yes	***	0.02961 to 0.07073
	Positive M vs negative F	0.03969	4.360	Yes	***	0.01567 to 0.06372
	Positive M vs positive F	0.04080	6.471	Yes	***	0.02416 to 0.05744
	Negative F vs positive F	0.001110	0.1160	No	**NS**	−0.02414 to 0.02636

Ab, antibody; CI, confidence interval; CRP, C-reactive protein; diff., difference; FT4, free thyroxine; HAV, hepatitis A virus; HBV, hepatitis B virus; NS, not significant; TSH, thyroid-stimulating hormone. *** $p < 0.001$.

Supplementary Table S8. Statistical analysis of inflammatory (CRP) and thyroid parameters (TSH and FT4) based on sex and HBV antibody status with the Bonferroni correction for multiple comparisons.

Parameter	HBV Ab and sex comparison	Mean diff.	t	*P* < 0.05?	Summary	95% CI of diff.
CRP (mg/dL)	Negative M vs positive M	0.008903	0.8916	No	**NS**	−0.01745 to 0.03526
	Negative M vs negative F	0.01457	1.087	No	**NS**	−0.02081 to 0.04994
	Negative M vs positive F	0.02434	2.176	No	**NS**	−0.005177 to 0.05386
	Positive M vs negative F	0.005662	0.4577	No	**NS**	−0.02699 to 0.03832
	Positive M vs positive F	0.01544	1.555	No	**NS**	−0.01076 to 0.04163
	Negative F vs positive F	0.009774	0.7317	No	**NS**	−0.02548 to 0.04503
TSH (μIU/mL)	Negative M vs positive M	0.07699	1.549	No	**NS**	−0.05421 to 0.2082
	Negative M vs negative F	−0.3358	5.033	Yes	***	−0.5120 to -0.1597
	Negative M vs positive F	−0.2541	4.563	Yes	***	−0.4010 to -0.1071
	Positive M vs negative F	−0.4128	6.702	Yes	***	−0.5754 to -0.2503
	Positive M vs positive F	−0.3311	6.700	Yes	***	−0.4615 to -0.2007
	Negative F vs positive F	0.08177	1.229	No	**NS**	−0.09377 to 0.2573
FT4 (ng/dL)	Negative M vs positive M	−0.001868	0.2786	No	**NS**	−0.01956 to 0.01583
	Negative M vs negative F	0.04906	5.451	Yes	***	0.02531 to 0.07281
	Negative M vs positive F	0.04673	6.223	Yes	***	0.02691 to 0.06655
	Positive M vs negative F	0.05093	6.130	Yes	***	0.02900 to 0.07286
	Positive M vs positive F	0.04860	7.293	Yes	***	0.03101 to 0.06619
	Negative F vs positive F	−0.002329	0.2597	No	**NS**	−0.02600 to 0.02134

CI, confidence interval; CRP, C-reactive protein; diff., difference; FT4, free thyroxine; HBV, hepatitis B virus; NS, not significant; TSH, thyroid-stimulating hormone. *** $p < 0.001$.

Supplementary Table S9. Statistical analysis of inflammatory (CRP) and thyroid parameters (TSH and FT4) based on sex and both HAV and HBV antibody status with the Bonferroni correction for multiple comparisons.

Parameter	HAV+HBV Ab and sex comparison	Mean diff.	t	*P* < 0.05?	Summary	95% CI of diff.
CRP (mg/dL)	Negative M vs positive M	0.001837	0.1676	No	**NS**	−0.02708 to 0.03076
	Negative M vs negative F	0.01457	0.8416	No	**NS**	−0.03110 to 0.06023
	Negative M vs positive F	0.02413	2.170	No	**NS**	−0.005202 to 0.05346
	Positive M vs negative F	0.01273	0.8802	No	**NS**	−0.02543 to 0.05089
	Positive M vs positive F	0.02229	3.872	Yes	***	0.007102 to 0.03748
	Negative F vs positive F	0.009561	0.6558	No	**NS**	−0.02891 to 0.04803
TSH (µIU/mL)	Negative M vs positive M	−0.007782	0.1252	No	**NS**	−0.1718 to 0.1562
	Negative M vs negative F	−0.3358	3.422	Yes	**	−0.5948 to -0.07691
	Negative M vs positive F	−0.3014	4.781	Yes	***	−0.4677 to -0.1351
	Positive M vs negative F	−0.3281	4.001	Yes	***	−0.5444 to -0.1117
	Positive M vs positive F	−0.2936	8.995	Yes	***	−0.3797 to -0.2075
	Negative F vs positive F	0.03449	0.4172	No	**NS**	−0.1836 to 0.2526
FT4 (ng/dL)	Negative M vs positive M	0.01480	2.521	No	NS	−0.0006905 to 0.03030
	Negative M vs negative F	0.04906	5.291	Yes	***	0.02460 to 0.07353
	Negative M vs positive F	0.04669	7.841	Yes	***	0.03098 to 0.06240
	Positive M vs negative F	0.03426	4.422	Yes	***	0.01382 to 0.05470
	Positive M vs positive F	0.03189	10.34	Yes	***	0.02375 to 0.04003
	Negative F vs positive F	−0.002370	0.3035	No	NS	−0.02298 to 0.01824

Ab, antibody; CI, confidence interval; CRP, C-reactive protein; diff., difference; FT4, free thyroxine; HAV, hepatitis A virus; HBV, hepatitis B virus; NS, not significant; TSH, thyroid-stimulating hormone. ** $p < 0.01$; *** $p < 0.001$.

Supplementary Table S10. The median and interquartile range (25%-75%) of inflammatory (CRP) and thyroid parameters (TSH and FT4) based on sex and both HAV and HBV antibody status.

		Male		Female	
		Negative	Positive	**Negative**	Positive
**Anti-HAV Ab**		(N = 1443)	(N = 3387)	(N = 782)	(N = 2175)
**CRP**	25% Percentile	0.0200	0.0300	0.0200	0.0300
	Median	0.0500	0.0600	0.0500	0.0600
	75% Percentile	0.1205	0.1200	0.1300	0.1150
**TSH**	25% Percentile	1.340	1.280	1.438	1.440
	Median	2.010	2.070	2.400	2.400
	75% Percentile	2.750	2.780	3.020	3.030
**FT4**	25% Percentile	1.180	1.160	1.120	1.130
	Median	1.280	1.270	1.230	1.230
	75% Percentile	1.400	1.390	1.350	1.350
**Anti-HBV Ab**		(N = 1443)	(N = 2492)	(N = 782)	(N = 1471)
**CRP**	25% Percentile	0.0300	0.0300	0.0200	0.0200
	Median	0.0600	0.0600	0.0400	0.0300
	75% Percentile	0.1300	0.1200	0.1000	0.0900
**TSH**	25% Percentile	1.340	1.260	1.438	1.530
	Median	2.010	1.980	2.400	2.400
	75% Percentile	2.750	2.720	3.020	3.000
**FT4**	25% Percentile	1.180	1.180	1.120	1.120
	Median	1.280	1.290	1.230	1.230
	75% Percentile	1.400	1.400	1.350	1.350
**Anti-HAV and anti-HBV Ab**		(N = 1443)	(N = 10,228)	(N = 782)	(N = 8311)
**CRP**	25% Percentile	0.0300	0.0300	0.0200	0.0200
	Median	0.0600	0.0500	0.0400	0.0400
	75% Percentile	0.1300	0.1100	0.1000	0.1000
**TSH**	25% Percentile	1.340	1.290	1.438	1.460
	Median	2.010	2.000	2.400	2.390
	75% Percentile	2.750	2.740	3.020	2.990

**FT4**	25% Percentile	1.180	1.160	1.120	1.120
	Median	1.280	1.270	1.230	1.230
	75% Percentile	1.400	1.390	1.350	1.350

Ab, antibody; CRP, C-reactive protein; FT4, free thyroxine; HAV, hepatitis A virus; HBV, hepatitis B virus; TSH, thyroid-stimulating hormone.
